# Factors Affecting the Adoption of Teledentistry Based on the Unified Theory of Acceptance and Use of Technology Model

**DOI:** 10.7759/cureus.46016

**Published:** 2023-09-26

**Authors:** Rayan Sharka, Lamer Sedayo, Samar Aldahwani, Laila Alyamani, Rahaf Budayri, Alaa Qari

**Affiliations:** 1 Dentistry, Faculty of Dental Medicine, Umm Al-Qura University, Makkah, SAU

**Keywords:** educational technology, technology acceptance, professional education, digital media, teledentistry

## Abstract

Objectives: The rise of teledentistry initiatives within the healthcare system is being recognized as healthcare institutions strive to decrease costs and enhance operational effectiveness. While previous research endeavors have examined various variables influencing the adoption of teledentistry, there exists a dearth of empirical studies that comprehensively investigate the success factors facilitating the acceptance of teledentistry. This research aimed to examine the factors that influence the behavioral intention of undergraduate dental students and interns to utilize teledentistry using the unified theory of acceptance and use of technology (UTAUT) as a theoretical framework.

Methods: A cross-sectional survey study was conducted in early 2023. An online survey was emailed to Bachelor of Dental Surgery (BDS) students from the fourth to sixth years and interns (N = 199) at a dental school in Saudi Arabia. A total of 187 students have responded (response rate: 93.9%). The survey comprised questions and scales that measured the UTAUT constructs of performance expectancy (PE), effort expectancy (EE), social influence (SI), facilitating conditions (FC), and behavioral intentions (BI). The data were analyzed using SPSS version 28 (IBM Corp., Armonk, NY).

Results: The findings of the study revealed that all UTAUT constructs had strong predictive power in relation to the BI in the decision to adopt teledentistry. Nevertheless, PE and EE were the salient factors. There was a statistically significant relationship between the UTAUT model and the BI, with the model explaining 60% of the variance in the BI (R2 = 0.606, P < 0.001).

Conclusion: The findings revealed that dental students' openness to teledentistry is influenced by their expectations for potential success, the level of work required, and the level of social pressure exerted on them. Thus, a number of different forms of support are required to boost teledentistry's uptake.

## Introduction

Nowadays, technology enables dental specialists to have access to a variety of media, including dental charts, photographs, radiographs, and written notes, which facilitates consultations with other dental specialists. Consequently, according to the International Association of Dental Research e-Oral Health Network (IADR/e-OHN), teledentistry can be referred to "as the uses of information and telecommunication technology to provide oral healthcare services between an oral healthcare provider and a patient or recipient or other health care providers who are separated by distance" [1].

Teledentistry has had a substantial impact by enhancing the accessibility of dental care providers, mitigating healthcare expenses, minimizing the possibility of infection, and optimizing patients' time utilization [2]. Prominent examples of teledentistry applications encompass dental screening, the identification of dental caries, the detection of both malignant and non-malignant lesions, and the management of patients with dental and temporomandibular problems [3,4]. There is more at stake for dental students, as teledentistry has proven to be a valuable tool for getting instruction from experienced dental professionals in detecting root canal orifices and managing dental trauma [5,6].

Even though earlier studies have verified the validity and effectiveness of teledentistry for improving dental care, its use in Saudi Arabia remains limited. In recent years, in line with the Kingdom’s Vision 2030, the government has made significant investments in the healthcare sector to improve access and quality of care for its citizens. This includes building new hospitals and clinics, increasing the number of healthcare professionals, and implementing digital health initiatives [7]. Despite this significant advancement, many issues, such as financial constraints, increased demand due to free services, poor accessibility to some medical centers, and a lack of adoption of the potential of digital health strategies, pose hurdles to the healthcare system [8]. In addition to the huge hurdles brought in by the COVID-19 pandemic [9]. In the face of these issues, there is a need for the adoption of teledentistry, which integrates digital technology into health systems and has, to some extent, the ability to combat the pandemic in the short term while strengthening health systems in the long run [10,11].

User acceptance of technology is a crucial factor in the effective adoption of this new healthcare modality. Prior studies have employed theoretical frameworks to understand factors and variables associated with users’ intentions to accept and utilize technological advances in medical care [12]. Despite the fact that the unified theory of acceptance and use of technology (UTAUT) model is a proactive and comprehensive approach that has been used in several countries and healthcare settings, little research has been conducted in the dental context, utilizing UTAUT to investigate dental students' acceptance and attitude toward the use of teledentistry. Also, the UTAUT model has been used to predict behavioral intention (BI) in a range of situations in dental education and services, including haptic virtual simulators, dental caries detection applications, and information system technologies in dental hospitals [13,14]. To the extent of our current understanding, there is a dearth of scholarly investigations pertaining to the use of a theory-driven approach for examining the perspectives of dental students and interns about the utilization of teledentistry. The following question was set in accordance with these presumptions: Which UTAUT construct has a salient influence on the overall decision to use teledentistry?

## Materials and methods

Ethical consideration

Ethical approval was obtained from Umm Al-Qura University’s Research Ethics Committee (Approval No.: HAPO-02-K-012-2022-11-1312).

Study design and sample population

This cross-sectional survey study was carried out in the second semester between January and March of 2023 at Umm Al-Qura University's Faculty of Dental Medicine in Saudi Arabia. A convenience sample of all dental students (undergraduates: Bachelor of Dental Surgery (BDS) cohorts, including BDS4 through BDS6, and interns) enrolling in the 2022-2023 academic year was contacted via email by administrators (program leads). Students in their pre-clinical years, i.e., BDS1 through BDS3, as well as students from other medical and health-related disciplines, were not invited. The determination of the sample size was conducted by considering the proportion of items (p) inside the scale relative to the total number of respondents (N). The optimal N:p ratios exhibit variation across different sources, ranging from three to 20 [15]. The present research has implemented the recommended average ground ratio of 10 individuals per item, as suggested by prior recommendations [16]. The sample size was estimated using the formula, N = 10 × 18 = 180, in accordance with the recommendation of Hair et al., with a confidence level of 95% and a sampling error of 5% [16].

Theoretical framework

**Figure 1:**
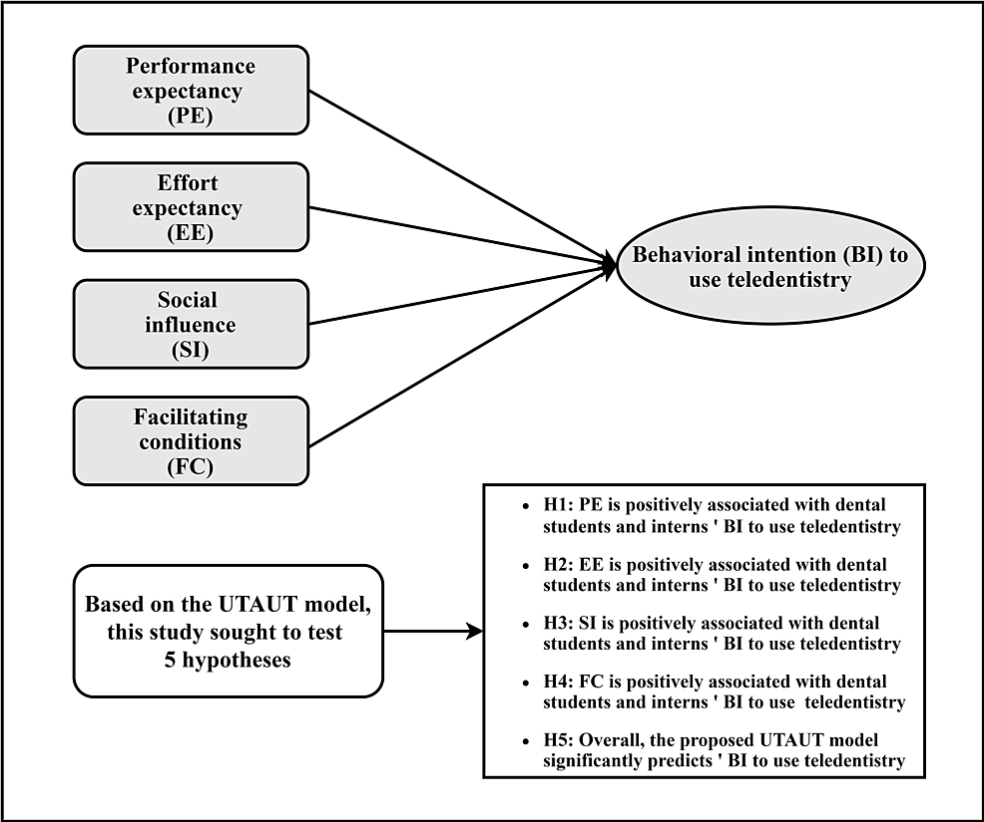
The proposed model and tested hypotheses. UTAUT: unified theory of acceptance and use of technology.

UTAUT can explain the acceptance and use of teledentistry among dental students [17]. UTAUT was originally created to understand the factors that influence employee acceptance and use of information technology [18]. Thus, this study utilized the UTAUT as a theoretical framework for the research. The main constructs of the model proposed to predict individuals’ BI to use technology, that is, performance expectation (PE), effort expectancy (EE), social influence (SI), and facilitating conditions (FC), play the role of independent variables (Figure 1). Contextually, PE is the extent to which a person believes that using teledentistry will improve job efficiency in the dental field. EE measures the level of ease related to the use of teledentistry. SI indicates how the individual perceives the opinions of others, such as peers or fellow workers, regarding the use of teledentistry. Lastly, FC is the degree to which an individual thinks that the dental institution or hospital and technical infrastructure operate to aid the use of teledentistry. The dependent variable in the utilized model was behavioral Intention (BI), which would assess dental students’ intention to use teledentistry in their future careers. Based on the UTAUT model, this study sought to test five hypotheses, as shown in Figure 1.

Questionnaire development

The questionnaire was accessed through a web-based survey (http://forms.microsoft.com) used to distribute the questionnaire, with a link to the questionnaire provided in the e-mail invitation. The questionnaire was divided into the following sections: section 1 included participants’ information sheet and consent form statements; section 2 included three demographic questions related to the student’s gender, age, and education level; section 3 contained the UTAUT model items that have been adopted by Alabdullah et al. to predict behavioral intentions to use teledentistry due to their validity and reliability [17]. It contains four segments measuring the UTAUT variables to predict behavioral intention (Figure 1). The responses were recorded on a five-point Likert scale ranging from 1 (strongly disagree) to 5 (strongly agree). Prior to the distribution of the main survey, a panel of five experts with specializations in digital media, teledentistry, and dental public health evaluated the instrument to ensure its content validity and reliability. This step was taken to ensure that the survey was clear and that its content accurately measured the intended constructs. Moreover, the final survey was attributed to a piloting phase with 10 dental students for the final review and testing of the questionnaire sections. In general, dental students thought the questionnaire questions were simple and straightforward.

Statistical analysis

Descriptive statistics were employed to assess dental students' and interns’ agreement with the UTAUT construct model. The reliability of the items' scales is assessed by the calculation of Cronbach's alpha (α). The Pearson correlation coefficient was used to assess the magnitude and direction of the linear relationship between the independent and dependent variables that were examined in the UTAUT model. To test hypotheses, inferential statistical methods such as simple and multiple regression analyses were used. A significance threshold of 0.05 was used for all analyses. The analysis was computed using the Statistical Package for the Social Sciences (SPSS) version 28.0 (IBM Corp., Armonk, NY).

## Results

Study participants

Email surveys were distributed to a total of 199 dental students and interns, and 187 completed responses were received and used in the data analysis, yielding a 94% response rate. The proportion of female respondents (52.9%) was just over half. The participants had a 23-year-old median age.

Descriptive statistics

Overall, the descriptive statistics results indicated that participants were quite agreeable with most of the items on the survey. Table 1 shows the mean composite score for each construct, averaged across the complete sample of dental students and interns. The range of ratings was between 3.442 and 4.055. The constructs with the highest scores were PE (M = 4.55, SD = 0.768), BI (M = 3.970, SD = 0.817), and EE (M = 3.945, SD = 0.671), following sequentially.

**Table 1:** UTAUT constructs and its measuring scale items. UTAUT: unified theory of acceptance and use of technology.

Factors	Items	Cronbach’s (α)	Mean (std)
Performance expectancy (PE)	PE1: Usefulness in managing dental practice. PE2: Increasing productivity. PE3: Saving time.	0.744	4.055 (0.768)
Effort expectancy (EE)	EE1: Clear and understandable. EE2: Easy to learn. EE3: Easy to use. EE4: Easy to operate.	0.752	3.945 (0.671)
Social influence (SI)	SI1: People recommend teledentistry. SI2: Friends recommend teledentistry. SI3: Institution recommends teledentistry.	0.732	3.442 (0.796)
Facilitating conditions (FC)	FC1: The resources to use teledentistry. FC2: Teledentistry know-how. FC3: Compatibility with other technologies. FC4: Presence of help. FC5: Assistance from the team.	0.648	3.621 (0.621)
Behavioral intention (BI)	BI1: Employing teledentistry in the future. BI2: Anticipating the use of teledentistry in the future. BI3: planning to use teledentistry in the future.	0.848	3.970 (0.817)

Assessment of reliability

The Cronbach's alpha coefficient was used to evaluate the internal consistency of the components that constitute the UTAUT. The Cronbach's alpha values observed in this study varied from 0.648 to 0.848, indicating a satisfactory degree of reliability (Table 1) [19].

Assessment of hypotheses

The findings revealed a highly significant positive association between PE and dental students' propensity to use teledentistry. The first analysis of the relationship between PE and BI revealed a significant positive correlation (r = 0.748, P < 0.001) (Table 2). PE explained 55% of the variance in BI (R2 = 0.557, F (1, 185) = 235.155, P < 0.001), thus supporting hypothesis 1 (Table 3). Secondly, the results revealed a significant positive correlation (r = 0.607, P = 0.001) between EE and the BI (Table 2). EE explained 36.8% of the variance in BI (R2 = 0.368, F (1, 185) = 107.698, P < 0.001), thus supporting hypothesis 2. Moreover, SI had a moderately significant positive association with BI (r = 0.54, P < 0.01). SI explained 29% of the variance in BI (R2 = 0.29, F (1, 185) = 78.365, P < 0.001), thus supporting hypothesis 3 (Table 3). Furthermore, the fourth hypothesis was supported, as FC showed a significant medium-positive association (r = 0.476, P < 0.001) with BI. FC explained 22% of the variance in the BI to use teledentistry (R2 = 0.22, F (1, 185) = 54.224, P < 0.001) (Table 3). Lastly, the final hypothesis was supported as the UTAUT model had significant predictive power for the intention to utilize teledentistry (R2 = 0.606, F (4, 182) = 70.084, P < 0.001). Moreover, it is worth noting that the UTAUT constructs were shown to explain 60% of the variation in BI (Table 3).

**Table 2:** Pearson correlation matrix between BI and each UTAUT construct. All correlations were significant at P < 0.001. UTAUT: unified theory of acceptance and use of technology; BI: behavioral intention; EE: effort expectancy; FC: facilitating conditions; PE: performance expectancy; SI: social influence.

Variables	PE	EE	SI	FC	BI
PE	1				
EE	0.672	1			
SI	0.506	0.497	1		
FC	0.516	0.619	0.546	1	
BI	0.748	0.607	0.545	0.476	1

**Table 3:** Predictive validity analysis. UTAUT: unified theory of acceptance and use of technology; BI: behavioral intention; EE: effort expectancy; FC: facilitating conditions; PE: performance expectancy; SI: social influence.

Hypotheses	UTAUT	R^2^	Std. error of the estimate	F	P	Hypothesis results
1	PE	55%	1.630	235.155	<0.001	Supported
2	EE	36%	1.953	107.698	<0.001	Supported
3	SI	29%	78.36	78.365	<0.001	Supported
4	FC	22%	2.161	54.224	<0.001	Supported
5	The model	60%	1.554	70.084	<0.001	Supported

## Discussion

The present study successfully identified four factors that had strong predictive capabilities in relation to the use of teledentistry technology. This finding provides empirical support for all of the research hypotheses.

PE had the most robust correlation, followed by EE, SI, and lastly, FC. The dental students' behavioral intention (BI) was shown to be significantly impacted by PE. One plausible reason for this outcome is that dental students expressed a desire to embrace teledentistry due to its perceived potential for enhancing their performance. They anticipated that this technology would significantly impact and improve dental practice and the entire clinical administration process. These findings are consistent with previous research that indicated PE to be the main indicator of BI usage of technology interventions in dentistry education when compared to other constructs [14].

Based on the results of this research, there is a significant correlation between EE and BI, suggesting that EE might serve as a valid indicator of dental students' acceptability and readiness to use teledentistry. The observed phenomenon might perhaps be explained by the notion that dental students attribute significant importance to the level of exertion necessary to use teledentistry technology. This reasoning is congruent with the findings of qualitative research among practicing orthodontists, which revealed that the ease of use and capabilities of digital dental technology in dental clinics are important motivators for technology adoption [20].

Moreover, SI and BI were significantly related (r = 0.54, P = 0.01). However, in contrast to previous research, a study used the UTAUT to predict dental students' intention to use haptic virtual simulators and discovered that EE was not a significant predictor [14]. This finding was supported by a previous empirical study that discovered that EE was not related to telehealth adoption by patients, clinicians, or agency leadership [21]. The predictive power of the relationship between FC and BI was found to be comparatively lower (R2 = 22%) when compared to the other components of UTAUT. According to these findings, dental students' opinions about the infrastructure and support for teledentistry may not have a significant impact on whether or not they decide to adopt it in the future. But in the dental care industry, FC still has a sizable impact on a professional's BI. A number of recent studies in the teledentistry context found comparable results. A previous multi-center study investigating teledentistry awareness and beliefs among dental students in the western region of Saudi Arabia discovered that 41.4% of students thought teledentistry required time and skills to operate, and 34% said a lack of present infrastructure may be a barrier to utilizing such technology [10]. In a similar vein, a cross-sectional study reported that 33% of Saudi dental professionals were concerned about teledentistry hardware and software, as well as the dependability of teledentistry technology [11].

All of the UTAUT components strongly predicted dental students' BI to utilize teledentistry in their future practice, according to the overall suggested model (Figure 1). PE, EE, SI, and FC accounted for 60% of the variation in the BI for teledentistry utilization. This result outperformed a previous study that used the same technology model in a teledentistry context [17] and was comparable with the initial suggested model by Venkatesh et al., which predicted 70% of the participants' BI, demonstrating an increasing UTAUT exploratory power and cumulative theory while maintaining a parsimonious structure [18].

Even though the survey received a high response rate of 94%, caution must be applied due to the convenience sampling from a single dental school in Saudi Arabia, which does not represent the target population as a whole. Also, to explore UTAUT scores longitudinally, test-retest repeatability as well as rigorous longitudinal investigations of one or more groups are required.

Moreover, the research did not examine the potential moderating effects of age, gender, and experience, which may have implications for the obtained findings. It is recommended that additional studies be carried out to further investigate the applicability of this model across other age cohorts. The examination of the acceptance of teledentistry in dental education among dental school directors, teaching personnel, and dental professionals may be deemed essential.

## Conclusions

This study contributes to a deeper understanding of the factors that affect the use of teledentistry in the dental profession in Saudi Arabia. The findings offer a fresh perspective and present an innovative approach to gathering more information. Also, this study offers valuable insights into the factors that impact the adoption of teledentistry among dental students. The adoption process is primarily influenced by performance expectation and effort expectancy, both of which play significant roles in shaping the process.
